# 

**Published:** 1958-04

**Authors:** 


### *Contents*

| | | Page |
|---|---|---|
| DISEASES IN SEARCH OF VIRUSES . | *Prof. C. H. Stuart-Harris* | 29 |
| PAIN RELATED TO THE TEETH AND JAWS . | *Prof. A. I. Darling* | 36 |
| SUPPORTIVE TREATMENT OF ACUTE COR PULMONALE DUE TO MASSIVE PULMONARY EMBOLISM | *Dr. E. A. W. Houghton* | 41 |
| ACCIDENTAL POISONING WITH THE ANTIHISTAMINE DRUG "HISTANTIN" . . . . | *Clinical Pathological Conference* | 45 |
| APOLOGY . . . . . . . . . | | 49 |
| CORRESPONDENCE . . . . . . . | | 50 |
| NOTES AND NEWS . . . . . . . | | 51 |
| APPOINTMENTS . . . . . . . . | | 53 |

